# Optimizing Dielectric Rod Antenna Performance with Spoof Surface Plasmon Polariton-Based Feeding Method

**DOI:** 10.3390/s24237543

**Published:** 2024-11-26

**Authors:** Rishitej Chaparala, Shaik Imamvali, Sreenivasulu Tupakula, Mohammad Aljaidi, Shonak Bansal, Krishna Prakash, Ali Fayez Alkoradees

**Affiliations:** 1 Department of Electronics and Communication Engineering, SRM University-AP, Guntur 522240, India; rishitej_chaparala@srmap.edu.in (R.C.); imamvali_shaik@srmap.edu.in (S.I.); 2 Department of Computer Science, Faculty of Information Technology, Zarqa University, Zarqa 13110, Jordan; mjaidi@zu.edu.jo; 3 Department of Electronics and Communication Engineering, Chandigarh University, Gharuan, Mohali 140413, India; shonakk@gmail.com; 4 Department of Electronics and Communication Engineering, NRI Institute of Technology, Eluru 521212, India; krris2k8@gmail.com; 5 Unit of Scientific Research, Applied College, Qassim University, Buraydah 51425, Saudi Arabia

**Keywords:** dielectric rod antenna, feeding element, impedance matching, metamaterial, plasmon, spoof surface plasmon polaritons (SSPPs)

## Abstract

This study investigates the use of spoof surface plasmon polaritons (SSPPs) as an effective feeding mechanism for antennas functioning within the extremely high-frequency (EHF) range. A novel method is proposed for feeding a dielectric rod antenna with SSPPs, featuring a simple design made from FR-4 material with a relative permittivity of 4.3. In contrast to traditional tapered dielectric rod antennas and their feeding configurations, this design shows promise for achieving a gain of up to 16.85 dBi with an antenna length of 7.6 λ_0_. By carefully optimizing the design, impedance matching and directional radiation characteristics were obtained at 7.3 GHz. Simulations were conducted using CST Microwave Studio to validate and evaluate the design’s performance. The enhanced gain, improved impedance bandwidth, and use of cost-effective materials such as FR-4 present a compelling case for adopting this design in future wireless communication technologies. Additionally, the remote sensing properties of the feeder can be utilized for concealed object detection, material characterization, and the analysis of the spectral properties of materials.

## 1. Introduction

Surface plasmon polaritons (SPPs) are electromagnetic waves that travel along the interface between a metal and a dielectric material at optical frequencies, enabling precise light manipulation at the nanometer scale. This capability offers significant advantages for nano-optical devices and sensitive optical measurements. Extensive research on nano-optical devices and optical waveguides [1,2,3,4] has revealed applications in super-resolution imaging, on-chip optical integrated circuits, biophotonics, microscopy, data storage, photovoltaics, graphene-based devices, photolithography, molecular sensors, photo catalysis, thin-film solar cells, and resonant waveguide gratings [5,6,7,8,9,10,11,12,13]. An intense and adjustable field interaction and amplification associated with transverse surface plasmon resonance (SPR) and cavity plasmon resonance (CPR) modes are noted [14]. A sensitive multichannel plasmonic sensor designed for refractive index and temperature sensing, operating across the visible to near-infrared spectrum [15], was studied. Researchers have investigated the application of SPPs at lower frequencies to support the development of integrated circuits and devices functioning in the microwave and terahertz ranges. Metals behave as perfect conductors at these frequency levels, preventing the support of SPPs. To overcome this limitation, plasmonic metamaterials [16,17,18,19] have been proposed to facilitate the realization of SPPs at reduced frequencies. The metallic surface was structured to enhance the interaction between the electric field and the metal. Because of their intrinsic three-dimensional geometries, these structures encounter considerable challenges in practical applications. Among the various SSPP structures, the ultrathin corrugated metallic strip featuring single and double periodic grooves is particularly noteworthy because of its simple planar design [20,21]. The independent operation of microwave SSPP devices poses challenges because of signal feeding and extraction inefficiencies, underscoring the importance of integrating them with conventional microwave circuits mainly made up of transmission lines. The efficient conversion of guided waves into SSPPs is essential. Various studies, such as those in references [22,23,24], have explored the conversion from transmission lines to plasmonic devices. Reference [22] employs corrugated grooves to create a conversion mechanism to facilitate conversion between CPW and SSPP components. Expanding on this concept, Ref. [23] modifies the conversion approach by placing grooves that extend outward from the inner conductor of the CPW instead of incorporating them internally. Both studies utilize the exponentially tapered Vivaldi slot line to facilitate the conversion process. Alternatively, Ref. [23] investigates a method that confines the SSPP wave between two oppositely oriented corrugated metallic strips, with electromagnetic energy coupled through a microstrip terminated by a circular metal plate. Regardless of these developments, existing SSPP devices still have limitations. In references [22,23], it was found that the Vivaldi slot remains essential, but its design parameters introduce added complexity. In [24], it was found that the requirement for a lower microstrip layer for coupling electromagnetic energy, along with an air hole at the input of the upper slot line, adds complexity to the design and makes it unsuitable for microwave-integrated circuits. In reference [25], the idea of spoof SPPs on ultrathin and flexible corrugated metallic strips was proposed and advanced to replicate SPPs within the microwave frequency range. The design approaches for both passive and active components converting to SSPP systems are described. These SSPPs provide advantages such as lightweight construction, flexibility, a low profile, and smooth integration with traditional microwave circuits. Various microwave systems that employ SSPPs are emphasized. Pendry et al. investigated the exceptional potential for engineering surface plasmons across a wide frequency spectrum. They showcased the capacity to manipulate and guide radiation at surfaces, enabling control over electromagnetic interactions over an extensive range of wavelengths. Utilizing surfaces and metals with high conductivity, they manipulated the dimensions and spacing of the holes to engineer specific surface plasmons. While they successfully produced surface plasmons, they were unable to generate SSPPs [26].

Another examination conducted by Hibbins et al. [17] investigated the pronounced concentration of the electric field at the boundary and examined the curvature of the light line, referred to as the dispersion curve. However, they did not observe the transmission of SSPP across the interfaces. Rusina et al. [27] developed grooved structures that facilitate SSPPs with defined width and depth, incorporated within a dielectric medium. Refined parameters of the structure enable the effective directing and trapping of light within THz fields. Gao Xu et al. [21] developed a thin dual-band plasmonic waveguide capable of supporting two specifically engineered SSPPs. Traditional techniques for feeding microstrip lines face difficulties like radiation losses, crosstalk, and mutual coupling. These issues can compromise signal integrity and restrict the overall performance of microwave systems. SSPP technology addresses this issue by enabling strong confinement achieved through wave manipulation on a sub-wavelength scale. Feeding based on SSPP technology presents numerous benefits: it features a reduced size compared to the microstrip designs, improves gain due to effective field confinement, and is easier and more economical to produce. SSPPs, which are engineered plasmonic metamaterials, replicate surface plasmon polariton (SPP) transmission in the microwave and terahertz frequency ranges by utilizing periodic subwavelength grooves or holes. Wenjuan et al. [28] suggested a transition structure that incorporates gradient-corrugated grooves with a base ground layer to modify microstrip lines to SSPP mode. This approach, known as “spoofing”, employs periodically arranged uniform-width stubs to create SSPP devices that imitate surface waves in the microwave and terahertz (THz) frequencies. Research [22,29,30] has demonstrated that a unit cell graph reveals a linear correlation between frequency and the propagation constant, together with amplified dispersion, as the groove height grows. Chaparala et al. successfully employed the SSPP structure by adjusting the groove width from 1 mm to 2 mm [31] while maintaining constant dimensions for the other aspects of the SSPP waveguide [28]. The expansion in groove width acted as an efficient feeding mechanism, resulting in a gain enhancement of about 0.83 dBi relative to the 1 mm design [31]. The waveguide was made of an FR4 dielectric substrate that measured 1.6 mm in thickness, along with a copper layer that was 0.035 mm thick. The FR4 used in the design had a dielectric constant of 4.3. These models can also be used in remote sensing applications.

With the advancement of wireless devices increasingly broadening their frequency range, the importance of antenna bandwidth has grown significantly. This trend has sparked a heightened interest in ultra-wideband (UWB) antennas, which provide a broad impedance bandwidth. Various UWB antennas are frequently utilized, such as log-periodic antennas, bow-tie antennas, spiral antennas, tapered dielectric rod antennas, and conventional transverse electric and magnetic (TEM) horn antennas. Of these, planar tapered dielectric rod antennas [32,33,34,35,36,37,38,39,40] are highly regarded for their potential to produce higher gain and increased power radiation when compared to other antennas of similar length. These antennas have excellent impedance matching, accommodate multiple elements, and minimize losses. This study introduces a patch-fed dielectric rod antenna inspired by research from Ghattas et al. This configuration shows enhanced gain capabilities compared to traditional tapered dielectric rod antennas. A variety of feeding techniques have been investigated for dielectric rod antennas in different applications. These methods encompass a patch-fed dielectric rod antenna designed for dual-band usage [32], a dielectric rod antenna connected to a rectangular waveguide for photo mixer-based terahertz sources [35], the optimal feeding of dielectric rod antenna arrays utilizing photonic crystal waveguides [34], and the excitation of both planar horn antennas and patch antennas using an SSPP waveguide [36,37,38,39].

Using the microstrip patch feeding method can lead to increased losses, potentially impacting the gain of the rod antenna and limiting its effectiveness for high-frequency applications. To mitigate this problem, we proposed the SSPP feeding technique, which facilitates high-frequency surface plasmon wave propagation through the use of periodic grooves at the metal–dielectric interface. This method allows for the transformation of quasi-transverse electric and magnetic waves from the microstrip line feeding into SSPP waves within the microwave frequency range. This study aims to introduce an innovative antenna-feeding structure by replacing the patch-fed antenna excitation with an SSPP-feeding element. Other structures, like a quarter-wavelength impedance transformation microstrip line or a microstrip coplanar waveguide (CPW), may also be employed. However, these methods are ineffective in lowering the rod antenna’s operating frequency from the millimeter waveband. The distinct advantage of SSPP feeding is its capacity to facilitate this frequency shift, proving to be more effective in modifying the operational bandwidth of dielectric rod antennas. The goal is to attain higher gain and enhanced impedance matching than those reported in prior studies [36,37,38,39,40,41,42].

## 2. Feeder Geometrical Properties

**Figure 1:**
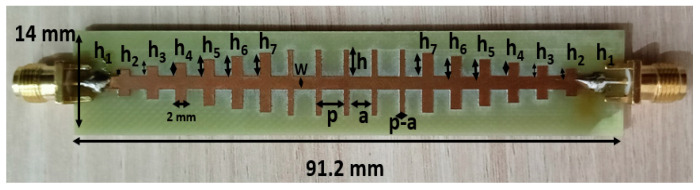
A fabricated device of a spoof surface plasmon polariton waveguide (h_1_ = 0.5 mm; h_2_ = 1 mm; h_3_ = 1.5 mm; h_4_ = 2 mm; h_5_ = 2.5 mm; h_6_ = 3 mm; h_7_ = 3.5 mm; p = 5 mm; a = 4 mm; p-a = 1 mm; W = 3 mm; h = 4 mm; SL = 14 mm; SW = 91.2 mm; and ST = 1.67 mm).

**Figure 2:**
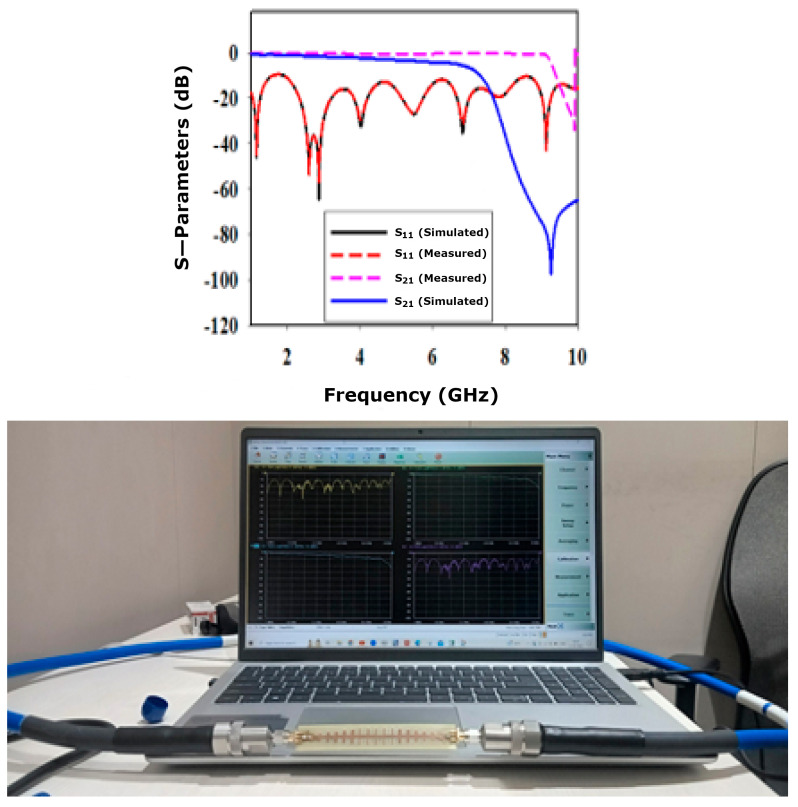
Simulated and measured characteristics of SSPP waveguide structure with measurement setup of SSPP waveguide.

This paper presents the SSPP waveguide structure and demonstrates its application as a feeding mechanism for the dielectric rod antenna. Figure 1 displays the fabricated SSPP waveguide structure, while Figure 2 presents the S-parameters, featuring both simulated and measured characteristics of the SSPP waveguide. The designed waveguide features gradient grooves with heights varying from h_1_ to h_7_ (0.5–3.5 mm) and dimensions of 91.2 × 14 × 1.67 mm^3^, with a groove height (h) of 4 mm, a pitch (p) of 5 mm, a groove width (a) of 4 mm, a gap (p-a) of 1 mm, and a width (W) of 3 mm. This arrangement enables the conversion of guided waves into SSPPs. In the simulated S-parameter characteristics shown in Figure 2, the reflection coefficient S_11_ is depicted by a black line, remaining below −10 dB across the frequency range of 1–10 GHz. This result indicates a strong level of optical confinement and impedance matching within the SSPP waveguide structure. The next section provides a detailed analysis of the patch-fed dielectric rod antenna.

The transmission coefficient |S21| of the simulated and measured characteristics can be observed at the resonating frequencies of 9.5 GHz and 10 GHz. The simulated and measured responses differ a bit due to fabrication tolerances, dimensional inaccuracies, and material properties, and surface roughness can affect the measured results compared to the simulated model. Additionally, connector losses, cable reflections, and calibration inaccuracies may introduce noise or cause shifts in the measured data that are not accounted for in simulations.

## 3. Numerical Simulation and Optimization

**Figure 3:**
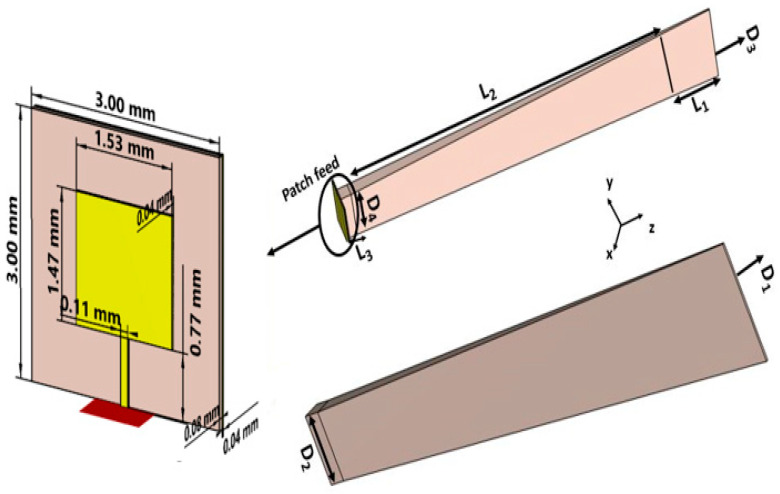
A simulated design of a patch-fed dielectric rod antenna representing the total rod length and diameter of tapering sections.

**Figure 4:**
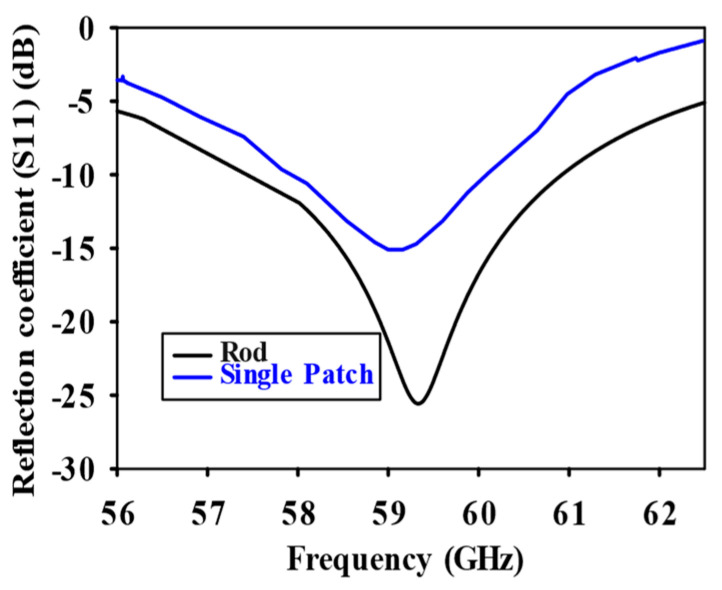
Reflection coefficient (S_11_) comparison for single patch and dielectric rod without SSPP feeding technique.

**Figure 5:**
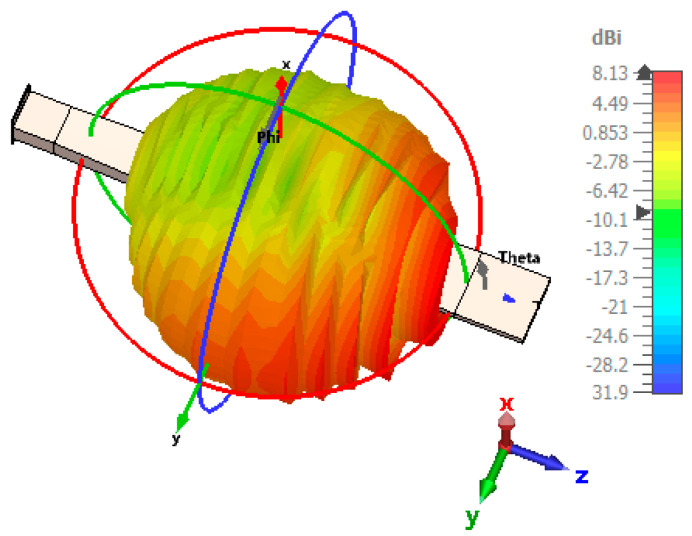
The gain of the patch-fed dielectric rod antenna at 59.3 GHz.

Figure 3 shows a schematic of the patch-fed rod antenna featuring a designated rod length. The design comprises a dielectric rod antenna and patch section made of FR4 material, which has a thickness of 1 mm and a relative permittivity of 4.3. The antenna is energized through a rectangular patch that measures 3 × 3 mm. L_1_, L_2_, and L_3_ correspond to the lengths of the dielectric rod antenna along the z-axis, while D_1_ and D_2_ refer to the measurements along the x-axis. To improve the gain, the upper and lower sections of the dielectric rod were tapered along the y-axis with lengths D_3_ and D_4_, where D_3_ is 0.08 λ_0_ and D_4_ is 0.28 λ_0_. This tapered section improves the gain of the antenna. The dimensions of the solid uniform sections of the dielectric rod antenna are provided in Table 1 and Table 2. The dimensions of each element were optimized to guarantee distinct incremental variations along the length of the central rod L_2_, spanning from 2λ_0_ to 7λ_0_. The objective of this process is to improve gain through optimization. The dimensions are fine-tuned to ensure that the rod antenna, powered by the patch, achieves its maximum gain. The antenna’s performance was carefully analyzed through simulations conducted within the frequency range of 56 to 65 GHz. Comprehensive simulations within the 56–65 GHz range confirmed impedance matching and maximized gain at 59.3 GHz. Figure 4 shows the comparison of the reflection coefficient between the single patch and the dielectric rod. Figure 5 highlights the gain of the patch-fed dielectric rod antenna, which is 8.13 dBi at 59.3 GHz.

The integration of the SSPP waveguide enabled the dielectric rod antenna to function effectively within the low-frequency band. Outstanding outcomes were obtained by combining the SSPP waveguide with a millimeter-wave band dielectric rod antenna operating in the 1–10 GHz range. The simulation results were recorded at a frequency of 7.1 GHz, maintaining the same dimensions as previously outlined.

**Figure 6:**
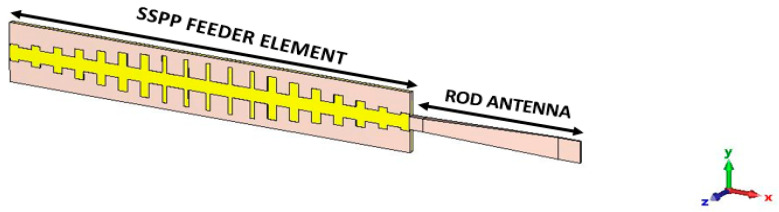
The novel feeding structure of the dielectric rod antenna with SSPP waveguide feeding.

**Figure 7:**
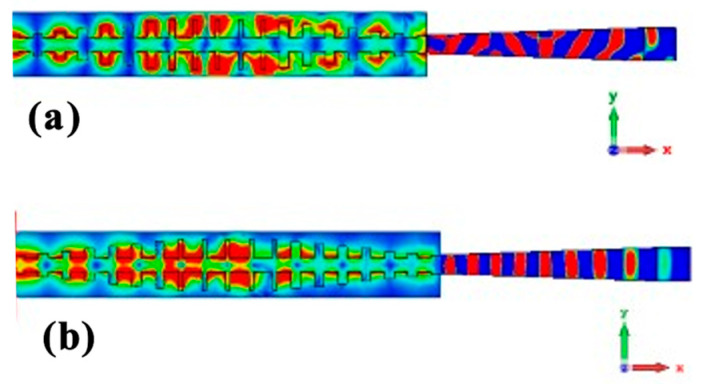
Distributed (**a**) E field and (**b**) H field radiation for designed SSPP-based dielectric rod antenna at 7.1 GHz.

In this configuration, the patch section was eliminated, allowing direct feeding of the rod antenna from the designed SSPP waveguide. Figure 6 depicts the link between the SSPP waveguide and antenna section. Feeding occurred through a single port located at the end of the SSPP feeding structure. The opposite end of the SSPP feeder was linked to the load comprising the rod antenna, as shown in Figure 6. Furthermore, Figure 7a,b show the distributed E field and H field radiation patterns of the SSPP-based feed for the dielectric rod antenna, simulated at a frequency of 7.1 GHz. The feeder generates confined electromagnetic waves capable of exciting a dielectric rod antenna. These waves travel along the x-axis, while the field is concentrated along the z-axis. The rod radiation is evident in Figure 7a,b. Figure 7b illustrates that the SSPP supports a magnetic field which is more effective in exciting the rod antenna compared to the electric field distribution.

## 4. Experimental Characterization and Results

**Figure 8:**
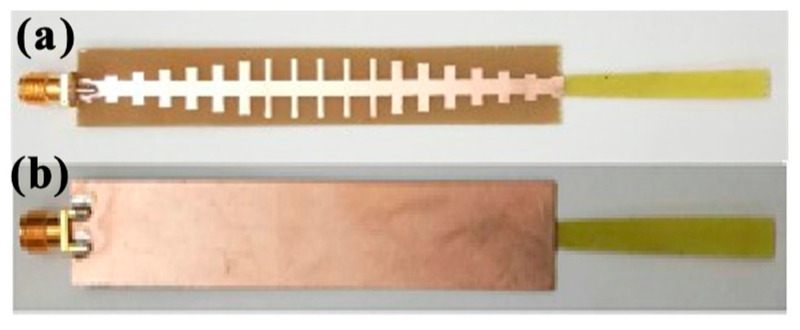
(**a**) Front and (**b**) back views of fabrication samples of the SSPP-fed dielectric rod antenna.

Figure 8 illustrates the fabricated sample displaying both the front and back sides of the proposed antenna. The fabricated samples were made with a PCB machine.

**Figure 9:**
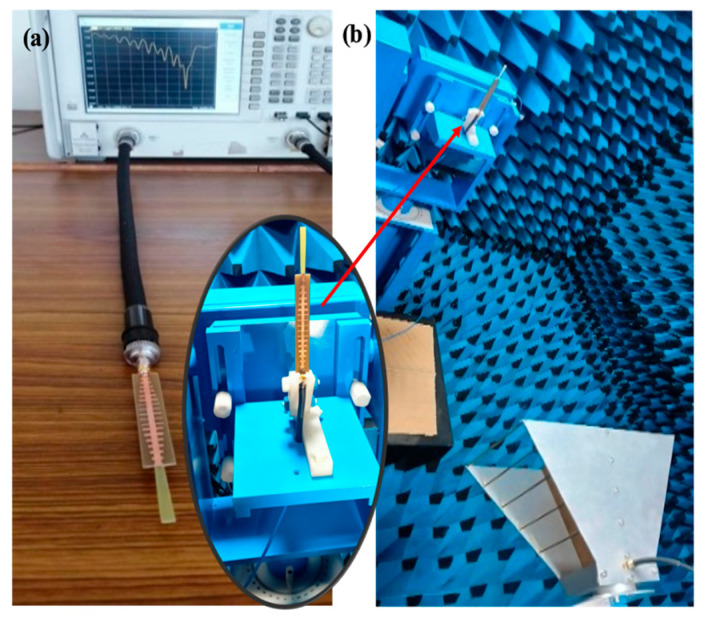
SSPP rod antenna. (**a**) Measurement of S-parameters with VNA. (**b**) Testing of SSPP rod antenna in anechoic chamber.

**Figure 10:**
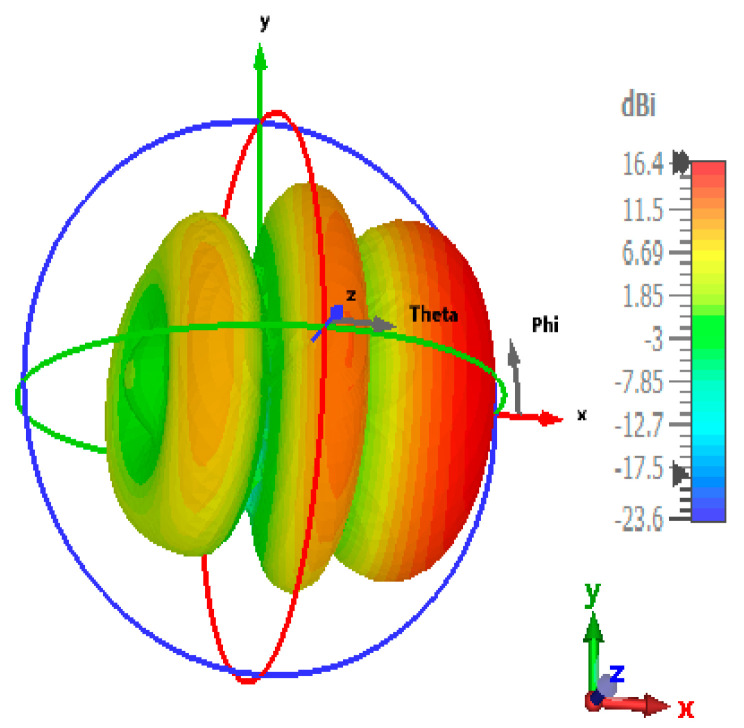
Gain of SSPP-fed dielectric rod antenna at 7.1 GHz.

**Figure 11:**
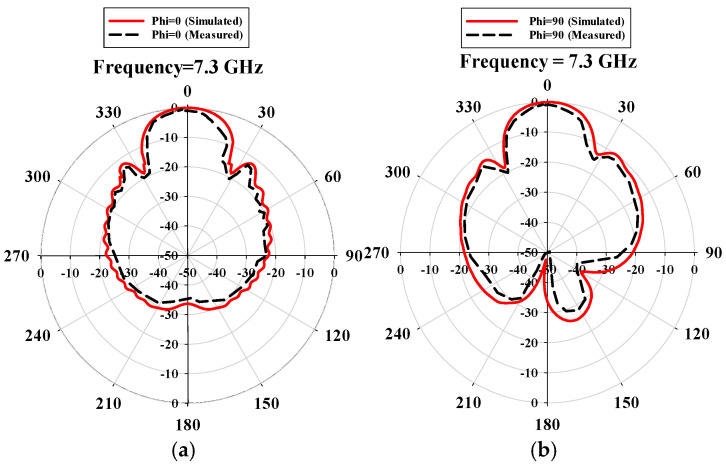
(**a**,**b**) represent the E and H plane patterns of the dielectric rod antenna with the SSPP feeding technique.

**Figure 12:**
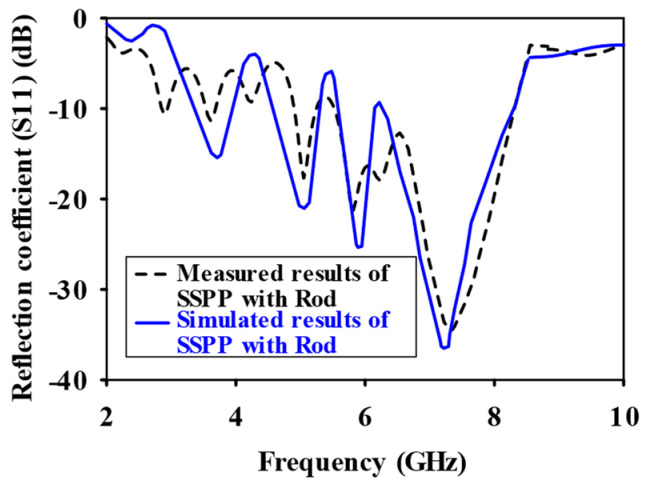
Reflection coefficient (S_11_) comparison for dielectric rod with SSPP feeding technique.

**Figure 13:**
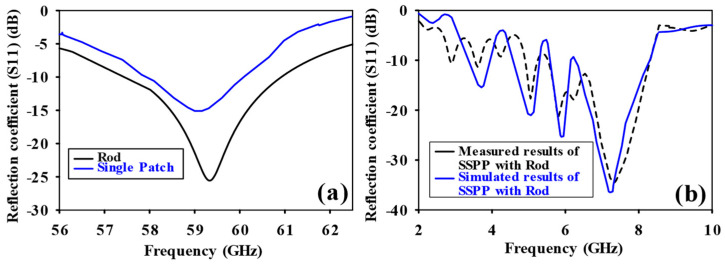
A comparison of significant results for the rod antenna (**a**) without SSPPs and (**b**) with SSPPs.

**Figure 14:**
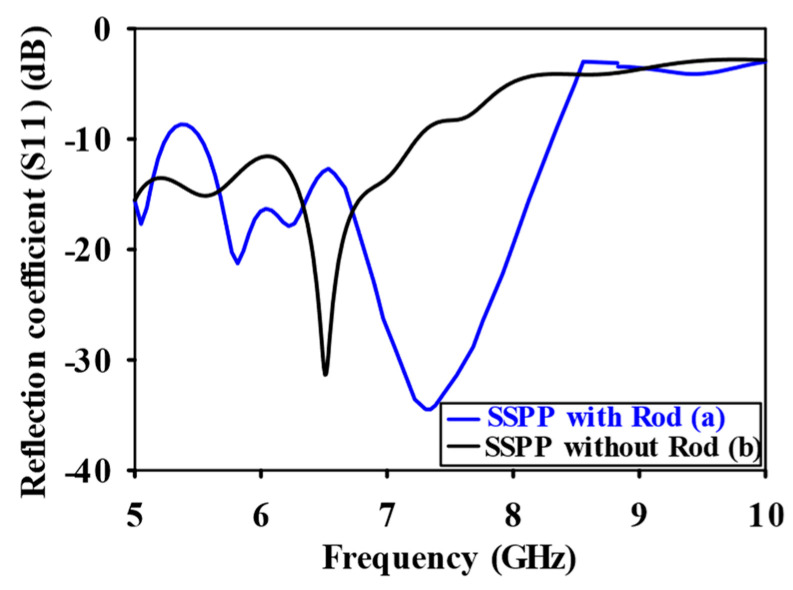
Comparison of impedance bandwidth curves of SSPPs (a) with rod and (b) without rod.

**Figure 15:**
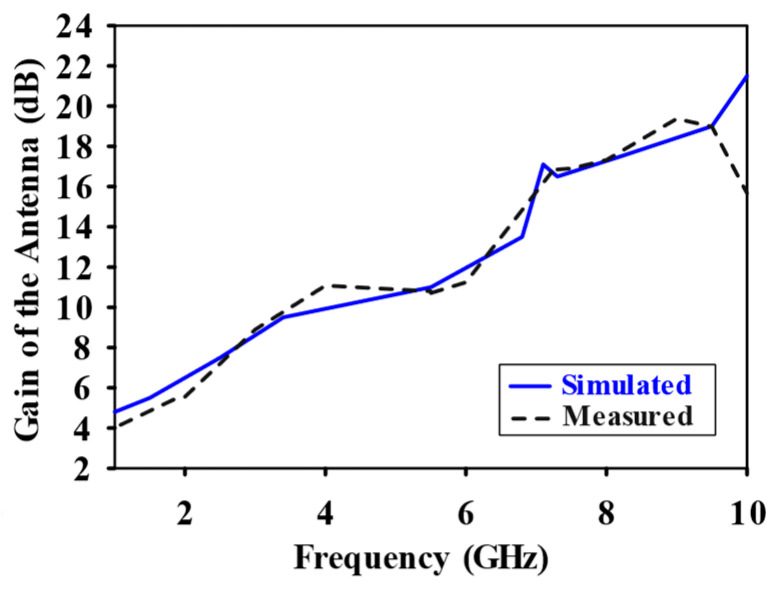
Gain vs. frequency plot for designed SSPP-based dielectric rod antenna for both simulated and measured data.

**Figure 16:**
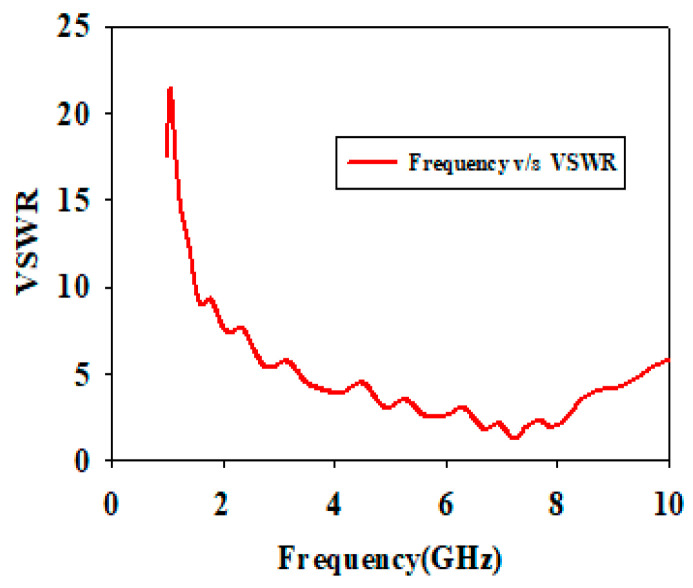
Simulated VSWR result of proposed configuration.

CST Microwave Studio was employed for simulation, while measurements were conducted using the Agilent N5247A vector analyzer (A.09.90.02), Keysight Technologies, Santa Rosa, CA, USA. Figure 9a,b illustrate the experimental setup for the proposed configuration. Figure 9a presents the initial reflection coefficient (S_11_) measurement of the SSPP-fed dielectric rod antenna, while Figure 9b shows the antenna installed in the anechoic chamber. Figure 10 shows that the simulation indicated a gain of 16.4 dBi at 7.1 GHz, whereas the actual measurements revealed a gain of 16.85 dB at 7.3 GHz, highlighting a significant peak at this frequency. The reference antenna gain (G_REF_) was assessed using an Amkom horn antenna (AMKOM Design Group Inc., Escondido, CA, USA), which operates from 1 to 18 GHz. The calibration of the vector network analyzer was conducted with a 3.5 SMA calibration kit, maintaining a 1.5 m distance between the AUT and the reference antenna. Figure 11a,b show the practical radiation patterns and the reflection coefficient at 7.3 GHz, and these results are compared with the CST Microwave Studio simulations. The E and H plane characteristics in Figure 11a indicate a main beam angle of approximately 30 degrees, which is consistent with the simulated patterns, confirming the effectiveness of the feeding technique in achieving high gain. Figure 12 displays the simulated and measured S11 of the design. Adjustments to the SSPP feeding structure involved changing the light line width from 2 mm to 3 mm, enhancing impedance matching. Although a slight frequency deviation was noted, the simulated (7.1 GHz) and measured (7.3 GHz) reflection coefficients were generally in agreement. Variations in the S parameters stemmed from differences in the loading effects between the simulated and measured setups, with measurement accuracy being influenced by the vector network analyzer’s precision. Both simulated and measured reflection coefficients remained below −10 dB within the 5 to 8 GHz bandwidth range. Figure 13 compares the patch-fed and SSPP-fed rod antenna configurations, showing a significant reduction in operating frequency from the range of 56–65 GHz to the range of 1–10 GHz. The decrease in the operating frequency of the dielectric rod antenna when using SSPP-based feeding is attributed to the unique properties of SSPPs. SSPPs emulate high-frequency surface waves, typically found in the optical or millimeter-wave range, allowing their propagation at lower microwave frequencies. This characteristic accounts for the antenna’s shift to lower operating frequencies with SSPP feeding. Figure 14 depicts S_11_ parameters for SSPP configurations, demonstrating that the SSPP-fed rod offers a broader impedance matching bandwidth than the SSPP alone, which acts only as a feeder. The curve representing the SSPP fed with the rod demonstrates a broader bandwidth for impedance matching compared to the curve without the rod. The difference in S_11_ for the SSPP without the rod is due to the SSPP acting solely as a feeder element. In this case, the electromagnetic waves propagate through the SSPP without a connected radiating component at the load section, resulting in an open circuit at the load, which produces a narrow S_11_ pattern. However, when the SSPP is connected to the dielectric rod antenna at the output load section, the load is effectively closed-circuited, leading to a broader S_11_ characteristic. Figure 15 shows the frequency-dependent gain curve for the SSPP-fed dielectric rod antenna, indicating a linear relationship with frequency. In Figure 16, the Voltage Standing Wave Ratio (VSWR) decreases to approximately 1.31 at 7.2 GHz, close to the theoretical value of 1.39. Table 3 summarizes improvements in the proposed configuration relative to other studies focusing on various feeding mechanisms across different frequencies, all aiming to enhance gain and impedance matching. The current SSPP configuration effectively confines electromagnetic waves, achieving optimal gain and decent impedance matching, as reflected in the results.

Other feeding methods, such as coaxial line and planar feeds, have notable limitations. Coaxial line feeds complicate modeling due to required substrate perforations, while planar feeds are better suited for arrays with significant interactions between elements and feed lines. Additionally, co-planar waveguide (CPW) feeding faces challenges due to a limited understanding of its practical applications within the microwave design community.

The advantages of the proposed design over the existing literature are as follows:

1. Simplified structure: The proposed structure offers a more straightforward design compared to a 1/4 impedance transformation microstrip line or a microstrip CPW structure [30,34,35,36,37,38,39,40,41,42,43,44].
2. Lower operating frequency: Due to the implementation of SSPP feeding, the dielectric rod antenna, which originally operated in the millimeter waveband, has been adapted to function within a lower frequency range of 1–10 GHz.
3. Enhanced gain: The proposed structure demonstrates improved gain over existing designs [30,34,35,36,37,38,39,40,41].
4. Improved impedance match: The reflection coefficient (S_11_) of the proposed design shows enhanced matching compared to the existing design [34,35,36,37,38,39,40,41].
5. Cost-effectiveness: It offers cost advantages in production, maintenance, and implementation, potentially leading to significant savings.

The limitations of the proposed design compared to the existing literature are as follows:

1. Feeder length: The rod antenna’s feeder in the proposed design made of SSPPs has a larger length compared to the antenna itself.
2. Complex field interaction: Differentiating between specific and nonspecific field interactions along the SSPP’s surface is challenging.

## 5. Conclusions

This study details the design, development, and analysis of an SSPP-fed dielectric rod antenna optimized for gain and impedance matching. The proposed structure allows for a smooth transition between conventional waveguides and plasmonic waveguides. Simulations reveal outstanding optical confinement of the waveguide across the millimeter-wave to microwave frequency range. This technology can serve as a remote sensor for low-frequency applications, enabling the detection of dielectric constants in various materials, the characterization of substances, the identification of gaseous mixtures, and food quality assessments, among other applications. This waveguide structure is effectively utilized to feed the dielectric rod antenna, which not only provides superior impedance matching compared to existing designs but also shows potential for integrating plasmonic waveguide circuits. As a result, researchers may explore the integration of multiple plasmonic devices for diverse applications following the techniques presented here.

## 6. Patents

Patent filed: 202341033912. Published on 23 July 2023.

## Figures and Tables

**Table 1:** Optimized measurements of the antenna.

L_1_	L_2_	L_3_	D_1_	D_2_
λ_0_	6 λ_0_	0.6 λ_0_	0.2 λ_0_	0.6 λ_0_

**Table 2:** Tapered rod section.

D_3_	D_4_
0.08 λ_0_	0.2 λ_0_

**Table 3:** Comparison of proposed work with existing methods.

Ref.	Feeding Source	(f_0_) GHz	Gain (dBi)	S_11_ (dB)	Antenna Length (mm)	Bandwidth (%)
[36]	SSPP	-	-	−29 dB	λ_0_/2	14.8
[37]	SSPP	4.5 GHz	8.43 dBi	−25 dB	5 λ_0_	6.5–10.4 GHz (46)
[38]	SSPP	30 GHz	6.8 dBi	−18 dB	6.08 × 4.51 mm	4.2–6.5 GHz (43)
[39]	SSPP	26.18 GHz	6.1 dBi	−28.8 dB	8.3 × 2 mm	27.9–30.5 GHz (8.82)
[40]	SSPP	27.2 GHz	15 dBi	−36 dB	λ_0_ + 1	27.5–32 GHz (55)
[41]	SSPP	11.5 GHz	6.8 dBi	−25 dB	6 λ_0_	7.5–9.3 GHz (21.35)
**[This work]**	**SSPP**	**7.2 GHz**	**16.4 dBi**	**−36.3 dB**	**7.6 λ_0_**	**5.4–8.2 GHz** **(39)**

## Data Availability

The data that support the findings of this study are available from the corresponding author upon reasonable request.
